# Inhibition of IDO leads to IL-6-dependent systemic inflammation in mice when combined with photodynamic therapy

**DOI:** 10.1007/s00262-020-02528-5

**Published:** 2020-02-28

**Authors:** Malgorzata Wachowska, Joanna Stachura, Katarzyna Tonecka, Klaudyna Fidyt, Agata Braniewska, Zuzanna Sas, Iwona Kotula, Tomasz Piotr Rygiel, Louis Boon, Jakub Golab, Angelika Muchowicz

**Affiliations:** 1 Department of Immunology, Medical University of Warsaw, Nielubowicza 5 Str., F Building, 02-097 Warsaw, Poland; 2 Department of Laboratory Diagnostics and Clinical Immunology of Developmental Age Medical, University of Warsaw, Warsaw, Poland; 3 Bioceros BV, Utrecht, The Netherlands; 4 Centre for Preclinical Research and Technology, Medical University of Warsaw, Warsaw, Poland; 5 Postgraduate School of Molecular Medicine, Medical University of Warsaw, Warsaw, Poland

**Keywords:** Photodynamic therapy, Indoleamine 2,3-dioxygenase 1, IL-6, Epacadostat

## Abstract

It was previously reported that the activation of antitumor immune response by photodynamic therapy (PDT) is crucial for its therapeutic outcome. Excessive PDT-mediated inflammation is accompanied by immunosuppressive mechanisms that protect tissues from destruction. Thus, the final effect of PDT strongly depends on the balance between the activation of an adoptive arm of immune response and a range of activated immunosuppressive mechanisms. Here, with flow cytometry and functional tests, we evaluate the immunosuppressive activity of tumor-associated myeloid cells after PDT. We investigate the antitumor potential of PDT combined with indoleamine 2,3-dioxygenase 1 (IDO) inhibitor in the murine 4T1 and E0771 orthotopic breast cancer models. We found that the expression of IDO, elevated after PDT, affects the polarization of T regulatory cells and influences the innate immune response. Our results indicate that, depending on a therapeutic scheme, overcoming IDO-induced immunosuppressive mechanisms after PDT can be beneficial or can lead to a systemic toxic reaction. The inhibition of IDO, shortly after PDT, activates IL-6-dependent toxic reactions that can be diminished by the use of anti-IL-6 antibodies. Our results emphasize that deeper investigation of the physiological role of IDO, an attractive target for immunotherapies of cancer, is of great importance.

**Electronic supplementary material:**

The online version of this article (10.1007/s00262-020-02528-5) contains supplementary material, which is available to authorized users.

## Introduction

Development of adaptive immune response is regulated by immunosuppressive mechanisms that are involved in the maintenance of tolerance to self-antigens as well as in the control of tissue damage and homeostasis. A balance between activation and inhibition of immune response is regulated at many levels by life-essential mechanisms and various cell types. Among others, indoleamine 2,3-dioxygenase 1 (IDO) was shown to be involved in the formation of a tolerogenic environment [1]. Moreover, in some types of cancer, IDO is considered to be engaged in the development of immunosuppressive microenvironment within the tumor and in the tumor-draining lymph nodes (TDLNs) [2]. The effects of IDO activity such as local depletion of tryptophan and production of kynurenines, cause growth arrest of effector T cells, loss of cytotoxic function and polarization into T regulatory lymphocytes (Treg). Additionally, it was reported that IDO-secreting cells can mediate apoptosis of T cell clones [3, 4]. IDO similarly to other amino acid degrading enzymes like arginase 1 (Arg1) can be induced during inflammation or anticancer therapy [5]. Secretion of interferon γ (IFN-γ) and tumor necrosis factor α (TNF-α) was shown to increase IDO expression in various types of myeloid cells, including monocytes/macrophages, neutrophils, dendritic cells as well as tumor cells. In many types of tumors, elevated expression of IDO correlates with poor prognosis of patients [6]. Therefore, IDO became a target for antitumor therapies and IDO inhibitors such as epacadostat, navoximod and indoximod are tested in clinical trials as mono- and combined therapies with other immunomodulatory drugs [7].

Development of strong inflammation is well described as a first and decisive event after photodynamic therapy (PDT) of cancer. PDT is a clinically approved, noninvasive cancer treatment involving generation of cytotoxic reactive oxygen species (ROS) that result from photosensitizer activation by light of appropriate wavelength. PDT leads to direct tumor cell death, disruption of vasculature followed by induction of acute inflammation [8, 9]. These events are associated with the release of various inflammatory mediators, recruitment and activation of innate immune cells and subsequent activation of a specific antitumor immune response. A great body of evidence indicates that the antitumor effects of PDT depend on the presence and activity of adaptive immunity [10].

Various immunosuppressive processes are also activated in response to PDT, including an increase in the number of Treg and production of anti-inflammatory cytokines, such as IL-10 or transforming growth factor β (TGF-β) [11]. Moreover, IL-10 and TGF-β mediate differentiation of CD4^+^ T cells into Treg and cause anergy of CD8^+^ T cells [12]. Importantly, inactivation of immunosuppressive mechanisms leads to the development of efficient PDT-mediated antitumor adaptive immune response [13].

An important role of immunomodulatory enzymes such as Arg1 or inducible nitric oxide synthases (iNOS) as well as myeloid cells in the shaping of PDT-treated tumor environment has been recently highlighted [14, 15]. In this study, we analyzed the expression of enzymes: IDO, Arg1 and iNOS to elucidate the immunosuppressive mechanism induced by PDT. We confirmed that PDT-mediated inflammation is associated with Treg induction, and we found that PDT triggers expansion of myeloid cells with elevated expression of IDO. Finally, we showed that the combination of PDT with IDO inhibitor (epacadostat) augments the IL-6-dependent acute inflammation. The antitumor efficacy of the treatment combining PDT and IDO inhibitor is effective but accompanied by systemic toxicity.

## Materials and methods

### Cell culture and reagents

Mammary carcinoma 4T1 cells were cultured in Dulbecco’s modified Eagle’s medium (DMEM) and E0771 in Roswell Park Memorial Institute (RPMI 1640) medium supplemented with heat-inactivated 10% fetal bovine serum (Invitrogen) and antibiotic/antimycotic solution (Sigma-Aldrich, A5955) under standard conditions (5% CO_2_, humidified incubator at 37 °C). Epacadostat and its analogue—INCB024360-analog (Medkoo Bioscience Inc), were prepared for administration as it was described by Koblish et al. [16]. Visudyne^®^ (Novartis), a liposomal formulation of verteporfin, was reconstituted as it was described before [17].

### Mice, tumor treatment and monitoring

Tumor cells were inoculated (5.0 × 10^4^ of 4T1 or 1.5 × 10^5^ of E0771 cells) into the second, left mammary fat pad of 8–12-week-old BALB/c or C57BL/6 female mice. The PDT was conducted on day 8th or 10th. Verteporfin was administered i.v., and PDT was performed as described previously [17]. Epacadostat was administered: orally twice a day from day 9th to 13th or on day 9th and 10th and subsequently from day 13th to 20th at a dose of 50 mg/kg or intratumorally once a day from day 9th to 16th at a dose of 100 mg/kg. The anti-IL-6 mAbs or appropriate isotype control was used at a dose of 100 µg per mouse and injected every second day, in 6 doses, starting from 1 day post-PDT. Tumors were measured as described before [17].

### Real-time PCR

Total RNA was isolated, and real-time PCR was done as previously described in detail [18]. The results were analyzed after amplification with LightCycler 480 Software 1.5 (Mannheim, Germany) and normalized for the content of the RPL32 as a housekeeping gene. The sequences of primers were as follows: IDO, forward-5′ GGTACATCACCATGGCGTATGTG-3′ and reverse-5′ TAAGACAGAATAGGAGGCA GGCC-3′; Arg1, forward-5′-GCAGTTGGAAGCATCTCTGG-3′ and reverse-5′-TCTACGTCTCGCAAGCCAAT; iNOS, forward-5′-GTCCTACACCA CACCAAACT-3′ and reverse-5′-CTCCAATCTCTGCCTATCCGT-3′; RPL32, forward-5′-TTAAGCGAAACTGGCGGAAAC and reverse-5′-TTGTTGCTCCCATAACCGATG-3′.

### IDO activity assay

Tumors and TDLNs were harvested and lysed in 0.5% NP-40. The enzymatic assay has been performed according to the method of Takikawa et al. [1] with some modifications. The reaction mixture (400 µl) contains: 1 M potassium phosphate buffer pH 6.5 (1 mM final concentration), 0.2 M sodium ascorbate (40 mM final concentration), 0.5 M methylene blue (20 µM final concentration), catalase (200 U/ml final concentration), l-tryptophan (400 µM final concentration) and 500 µg of protein. All needed chemicals were purchased from Sigma-Aldrich. After 60 min of incubation at 37 °C, the reaction was terminated by the addition of 80 µl of 30% TCA. To convert *N*-formylkynurenine to kynurenine, the reaction was carried out at 60 °C for 15 min, followed by centrifugation (10,000 × *g*, 20 min). Kynurenine was quantified by the addition of 200 µl of 2% Ehrlich’s reagent in a glacial acetic acid to an equal volume of sample supernatant. The activity of IDO was defined as the concentration of kynurenine that was generated during 60 min of enzymatic reaction. The kynurenine concentration was revealed as absorbance, measured at 480 nm (ASYS UVM 340, Biochrom) and shown on the graph as a percentage of controls.

### Cell isolation

In order to obtain a single cell suspension, the tumors and TDLNs were incubated with collagenase IV (Sigma-Aldrich, C5138) and DNAse (Sigma-Aldrich, DN25) in IMDM medium (Invitrogen, 12440053) and subsequently forced through a 100-μm strainer. CD11b^+^ cells were isolated from tumor cell suspension by positive selection on magnetic beads according to the manufacturer’s instruction (EasySep #18000, Stemcell Technology). Spleens were mechanically disrupted through a 70-μm cell strainer.

### White blood cells analysis

The smears of the blood were stained with the May–Grunwald–Giemsa method and properly air dried. Next, detailed cell morphology was assessed under the light microscopy with 100 × oil immersion objective. In the prepared smear, the percentages of the various population of white blood cells were counted.

### Co-culture proliferation assay

Splenocytes were subjected to negative selection using magnetic beads (EasySep™ Mouse T Cell Enrichment Kit). Subsequently, CD3^+^ splenocytes were stained with CellTracker™ Violet BMQC Dye (Thermo Fisher) for 20 min in 37 °C and washed two times. Next, CD11b^+^ cells and T cells were seeded onto previously coated with anti-CD3 antibody (145-2C11, eBioscience) 96-well plate in 2:1 ratio. Then, cells were stimulated with anti-CD28 (145-2C11, eBioscience) for three consecutive days. Subsequently, T cells were stained and proliferation of CD8^+^ (53-6.7, eBioscience) and CD4^+^ (RM4-5, eBioscience) cells was evaluated with FACSCanto II using Diva software (Becton Dickinson, Franklin Lakes, New Jersey, USA).

### Staining and flow cytometry

Cells were stained with Zombie NIR™ Fixable Viability kit (BioLegend, 423106) according to the manufacturer’s protocol and blocked with anti-CD16 mAbs. For surface markers, subsequent antibodies were used: anti-CD45.2-V500 (104, BD Bioscience), anti-CD11b-FITC (M1/70, eBioscience), anti-Ly6C-PerCp-Cy7 (AL27, BD Bioscience), anti-Ly6G-APC (1A8, BioLegend), anti-CD3-V450 (17A2, eBioscience), anti-IL4R-PE (552509, BD Bioscience). For intracellular staining, after the cells were fixed and permeabilized with Cytofix/Cytoperm (554722, BD Bioscience), the following antibodies were applied: anti-IDO-eF660 (Mido-48, eBioscience) and anti-Arg1-PE (IC5868P, R&D). In order to analyze the Treg population, the Mouse Phenotyping Kit (560767, BD Bioscience) was used. Cells, resuspended in FACS flow buffer, were analyzed on FACSCanto II using Diva software. The cytokine concentration was measured in mouse serum, separated from the blood collected from the cheek vein. Serum was stained with BD™ Mouse Inflammation Kit, Cytometric Bead Array (552364, BD Bioscience).

### Statistical analyses

Data were analyzed with GraphPad Prism 6, and differences were calculated for significance by Mann–Whitney U test. The survival rate of animals was analyzed by log-rank survival test.

## Results

### PDT triggers functional and phenotypic changes in CD11b^+^ tumor-associated cells

**Fig. 1:**
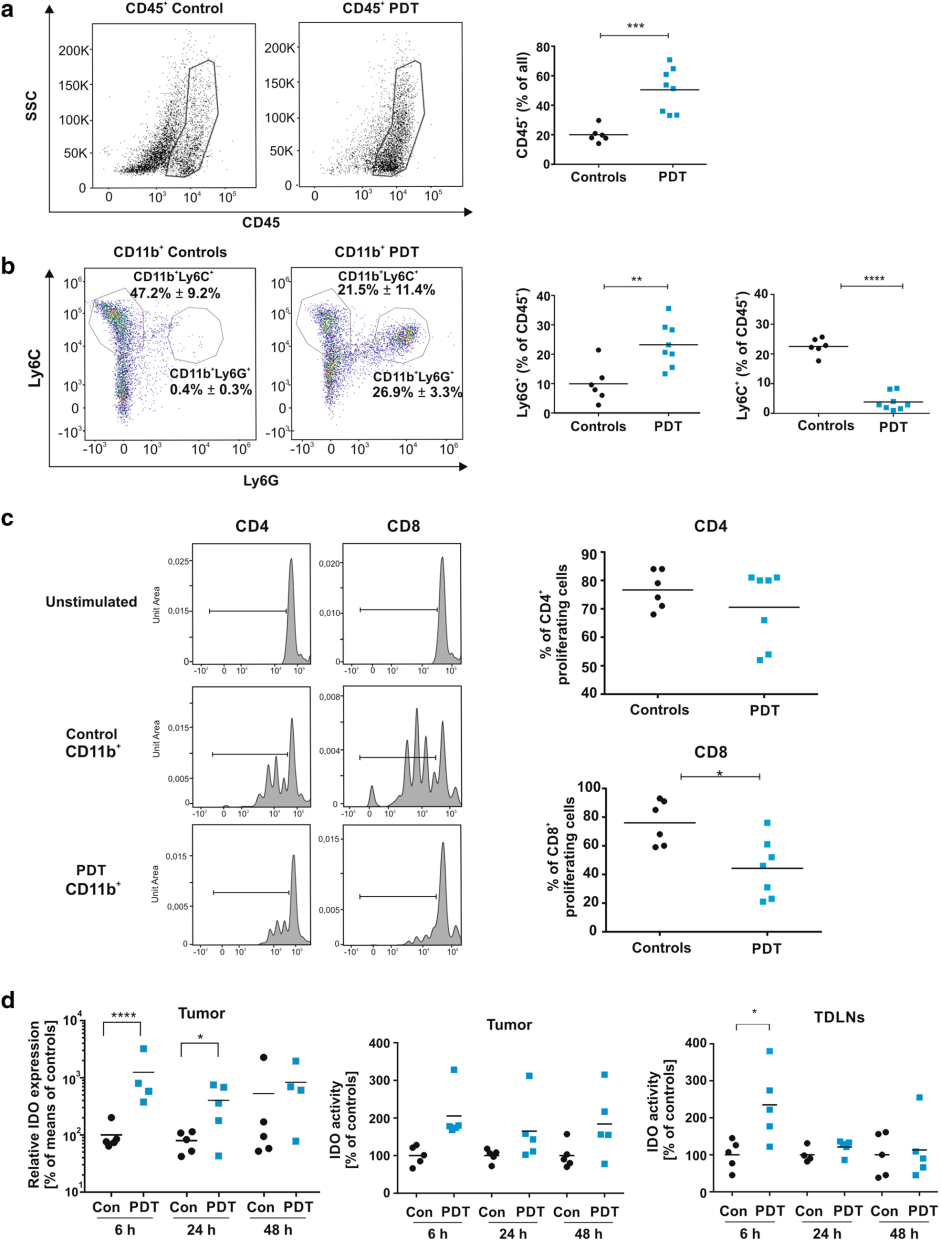
Influence of PDT on tumor-associated immune cells. The percentage of CD45^+^ cells (**a**), Ly6C^+^ and Ly6G^+^ cells shown as a parent of CD11b^+^ (**b,** dot plots, left) and CD11b^+^Ly6C^+^ and CD11b^+^Ly6G^+^ presented as a parent of CD45^+^ (**b,** graph, right) in E0771 tumors collected from control and PDT-treated group 3 days post-treatment and analyzed by flow cytometry. Data present individual values with the means (bars), *n* = 6–8; **P < 0.01; ***P < 0.001; ****P < 0.0001. **c** Proliferation rate of CD4^+^ and CD8^+^ lymphocytes after incubation with tumor-derived CD11b^+^. CD3^+^ T cells from spleen of donor mouse were stained with cell tracker dye and incubated with tumor-derived CD11b^+^ cells (control and PDT-treated group 3 days post-treatment) for three consecutive days, counterstained with anti-CD4-FITC and anti-CD8-PerCp-Cy5 antibodies and analyzed by flow cytometry. The data are presented as representative histograms and on the graphs as % of proliferating cells, individual values with the means (bars), *n* = 6–8; *P < 0.05. **d** Expression and activity of IDO. IDO expression was measured in E0771 tumors 6, 24 and 48 h post-PDT. mRNA levels were determined using real-time PCR. Data present  % of means of controls in experimental groups, *n* = 4–5; *P < 0.05. Activity of IDO enzyme was evaluated in E0771 tumors and TDLNs 6, 24 and 48 h post-PDT. Data present  % of controls in experimental groups, *n* = 4–6; *P < 0.05; **P < 0.01. All experiments were repeated at least 2–3 times, and the representative results are shown

Tumor-residing myeloid cells are considered to be immunoregulatory and were reported to suppress multiple effector pathways of T and NK cells, including their cytotoxic activity, proliferation and cytokine secretion [19]. PDT is cytotoxic toward tumor cells as well as tumor stromal cells including endothelial cells and tumor-infiltrating leukocytes [20, 21]. However, soon after PDT the tumor bed becomes densely infiltrated by myeloid cells and these newly infiltrating cells were reported to contribute to tumoricidal effects of PDT [22]. Indeed, we observed that PDT increases the percentage of CD45^+^ cells in 4T1 tumors (Fig. 1a). Flow cytometry analysis of tumor-infiltrating CD45^+^CD11b^+^ cells (Supplementary Fig. 1 for gating strategy) revealed that PDT mainly leads to a significant increase in the percentage of Ly6G^+^ granulocytic myeloid cells (Fig. 1b).

**Fig. 2:**
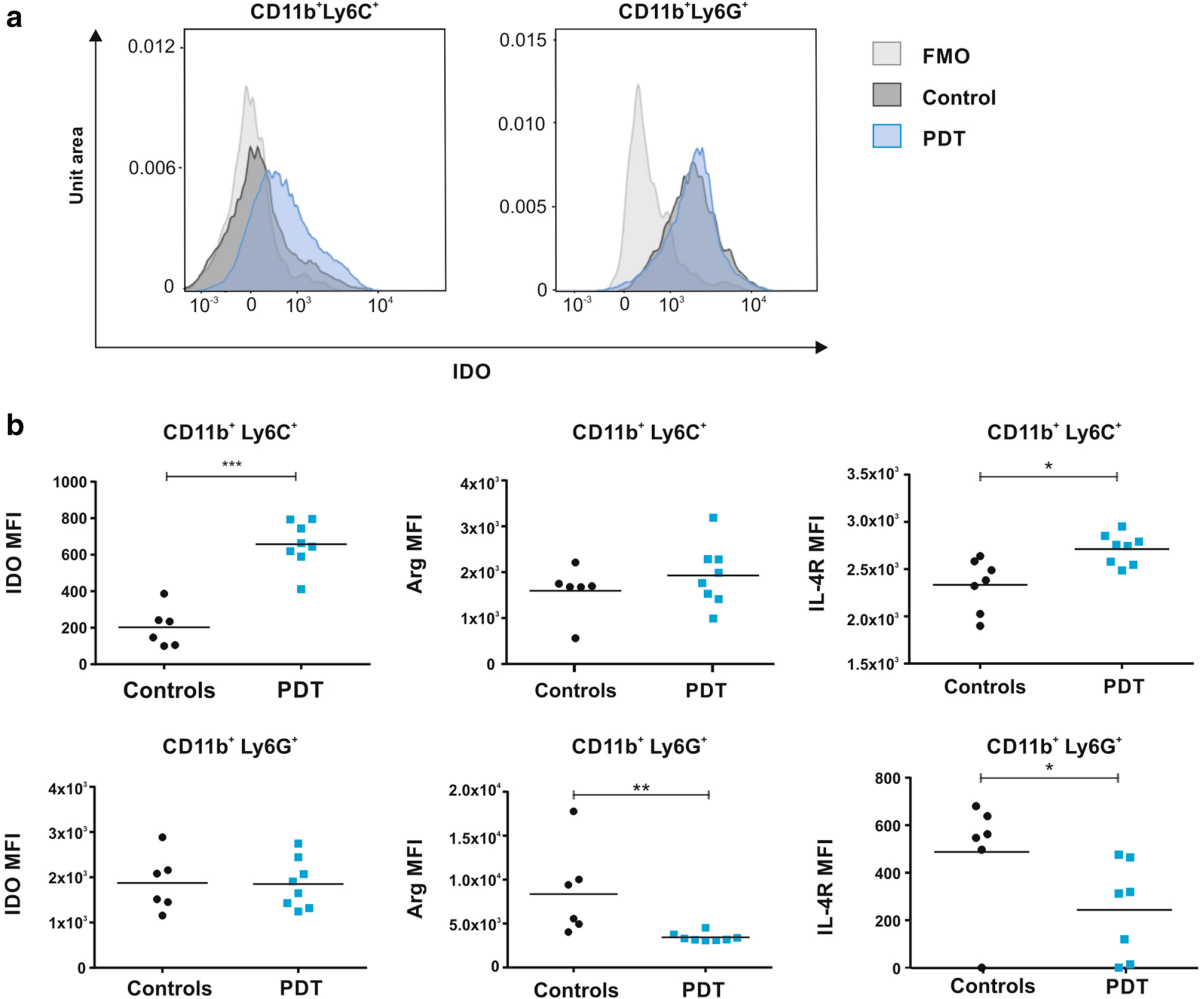
Influence of PDT on CD11b^+^ tumor-associated cells. **a** IDO expression in CD11b^+^Ly6C^+^ and CD11b^+^Ly6G^+^ cells. **b** Mean fluorescence intensity (MFI) of IDO, Arg-1 and IL-4R expression measured by flow cytometry in tumor-derived CD11b^+^Ly6C^+^ (upper panel) and CD11b^+^Ly6G^+^ (lower panel) cells isolated from control and PDT-treated tumors 3 days post-treatment. Data present individual values with the means (bars), *n* = 6–8; *P < 0.05; **P < 0.01; ***P < 0.001 CD45^+^CD11b^+^Ly6C^+^ and CD45^+^CD11b^+^Ly6G^+^ cells, collected from control and PDT-treated tumors 3 days post-treatment and analyzed by flow cytometry. The experiment was repeated at least 2 times, and the representative results are shown

We hypothesized that myeloid cells infiltrating PDT-treated tumors and encountering cells that underwent immunogenic cell death [23] might have an increased immunostimulatory potential as compared to myeloid cells before treatment. Unexpectedly, CD11b^+^ cells, magnetically isolated from PDT-treated tumors, turned out to cause stronger suppression of the splenic CD4^+^ and CD8^+^ T cell proliferation as compared with cells isolated from control tumors (Fig. 1c). The immunosuppressive environment in tumor bed was confirmed by increased expression of Arg1, 24 and 48 h after PDT, and iNOS 24 h post-PDT (Supplementary Figure 2a). Additionally, real-time PCR revealed a rapid and very strong (over tenfold over controls) increase in IDO expression in PDT-treated tumors (Fig. 1d, left), which translated into over twofold increase in IDO enzymatic activity (Fig. 1d, middle). A transient increase in IDO activity was also observed in TDLNs (Fig. 1d, right and Supplementary Fig. 2B). Flow cytometry showed that granulocytic CD11b^+^Ly6G^+^ cells express approximately 10 times higher levels of IDO in comparison with monocytic CD11b^+^Ly6C^+^, suggesting that granulocytic cells are the major source of IDO within the tumors. Importantly, the level of IDO, as well as the level of the IL-4R in monocytic cells, significantly increased after PDT suggesting the immunosuppressive properties of this population (Fig. 2b). Nevertheless, the major increase in IDO levels in tumors may result from the augmented infiltration by granulocytic CD11b^+^Ly6G^+^ cells into the tumor bed. Granulocytic CD11b^+^Ly6G^+^ cells express relatively lower levels of Arg1 (Fig. 2b, middle) and IL-4R (Fig. 2b, right) after PDT with verteporfin, indicating that IDO might be involved in myeloid cell-mediated suppression of T cell proliferation after PDT.

### Inhibition of IDO potentiates antitumor effects of PDT but is associated with systemic inflammation

**Fig. 3:**
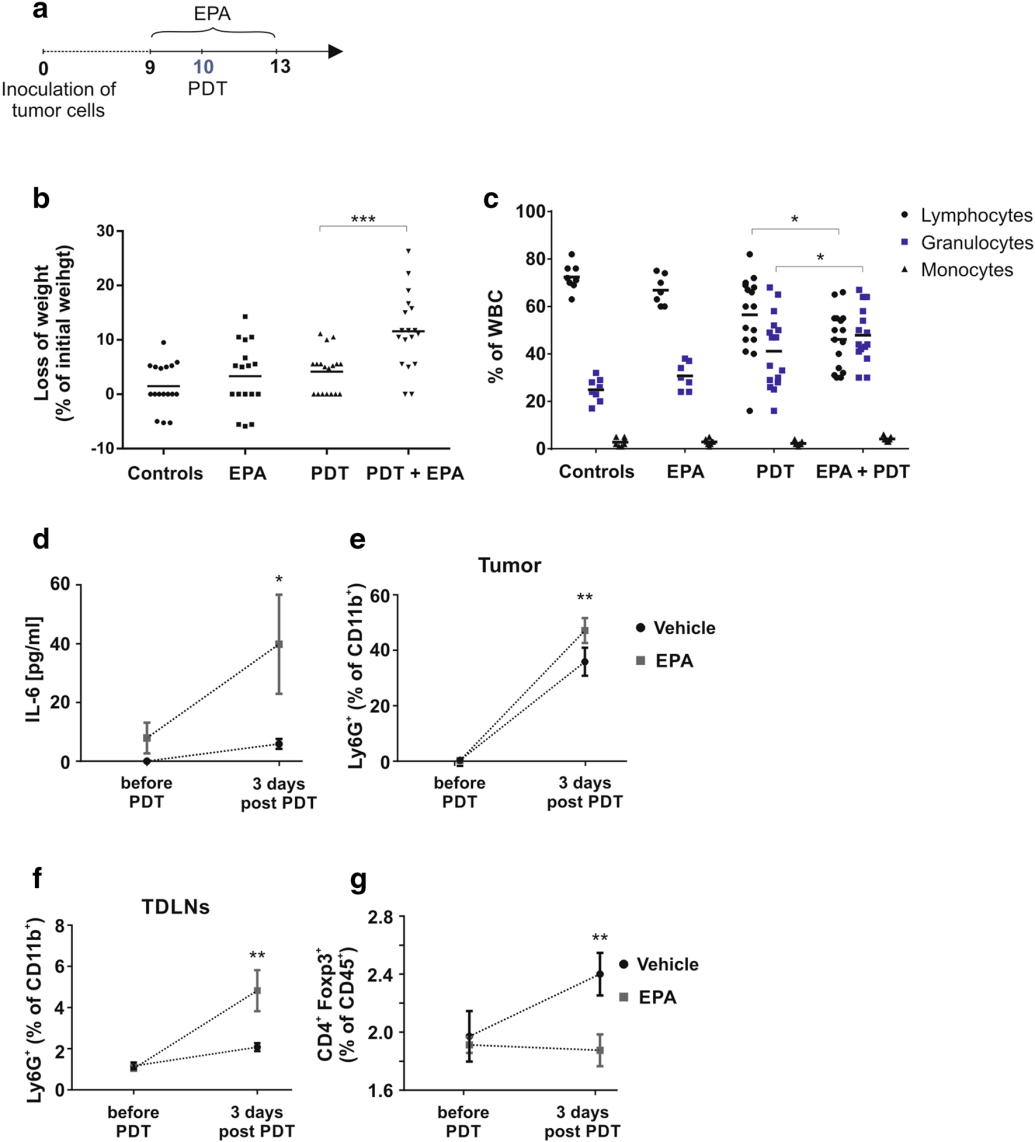
PDT combined with IDO inhibitor leads to systemic lethal reaction. **a** Detailed experimental scheme. **b** Loss of weight (day 13th of experiment) presented as percentage of initial weight. Data present individual values with the means (bars), from three independent experiments, *n* = 17; *P < 0.05; ***P < 0.005. **c** Percentage of white blood cells. Blood was collected 3 days post-PDT for all experimental groups, blood smears were prepared and lymphocytes, granulocytes and monocytes were counted. Experiment was repeated 2 times, and data are presented as percentage of white blood cells, *n* = 9; *P < 0.05. **d** Amount of IL-6 in serum collected from mice before and 3 days after PDT, IL-6 was analyzed by flow cytometric bead array and presented in pg/ml. Data present mean values ± SEM; *P < 0.05, *n* = 9. **e**, **f** The percentage of immunological cells CD11b^+^Ly6G^+^ in E0771 tumors (E) and TDLNs (F) collected from mice treated or untreated with IDO inhibitor before and after PDT, analyzed by flow cytometry. Data present mean values ± SEM; **P < 0.01, *n* = 6–8. The experiment was repeated at least 2 times, and the representative results are shown. **g** Percentage of Treg in TDLNs of mammary E0771 tumors. TDLNs were collected before and 3 days post-PDT; cells were analyzed by flow cytometry. Graph presents CD4^+^Foxp3^+^ as percentage of CD45^+^ immunological cells. Data present mean values ± SEM; **P < 0.01, *n* = 9

To investigate whether IDO inhibition might improve the antitumor efficacy of PDT, by mitigating its suppressive effects on T cells, we combined PDT with IDO inhibitors: epacadostat (EPA, INCB024360) and its structurally related analogue (4-amino-*N*-(3-chloro-4-fluorophenyl)-*N′*-hydroxy-1,2,5-oxadiazole-3-carboximidamide) in murine E0771 breast carcinoma model. Both compounds are potent IDO inhibitors with the same IC_50_ (10 nM) determined with the recombinant enzyme [16]. Treatment with EPA at a dose of 50 mg/kg twice daily was started 24 h before PDT and was planned to be continued for the next 10 days. While EPA administration was well tolerated in control mice, we had to discontinue the experiment after eight doses of EPA administration (day 4 of the treatment protocol, Fig. 3a) due to strong toxicity observed in mice that were also treated with PDT. Mice rapidly lost the weight (Fig. 3b), and 20% of animals died in the first 4 days after the PDT illumination. Similar toxicity of the combined treatment was observed in 4T1 tumor model in BALB/c mice (Supplementary Fig. 2C, D). Considering that both PDT and EPA exert multiple immunoregulatory activities, we used flow cytometric beads array to measure serum cytokines. Serum IL-6 concentration was elevated after PDT, and the addition of EPA further increased IL-6 (Fig. 3d). The increase in serum IL-6 concentration was associated with marked neutrophilia in PDT-treated mice, which was significantly more pronounced in mice treated with PDT and EPA (Fig. 3c). The higher neutrophil counts in peripheral blood were associated with the massive tumor and TDLNs neutrophil infiltration (Fig. 3e, f), indicating systemic inflammation developed after combined treatment. It was previously reported that PDT increases the number of CD4^+^FoxP3^+^ Treg [11]. We observed that the percentage of TDLN Treg increased after PDT, and this increase was significantly suppressed by the addition of EPA (Fig. 3g), suggesting that IDO induction might be associated with the expansion of these regulatory cells after PDT. Altogether, these observations indicate that IDO inhibition after PDT induces systemic inflammation that leads to exaggerated toxicity.

### Mitigation of systemic toxicity is associated with reduced antitumor effects

**Fig. 4:**
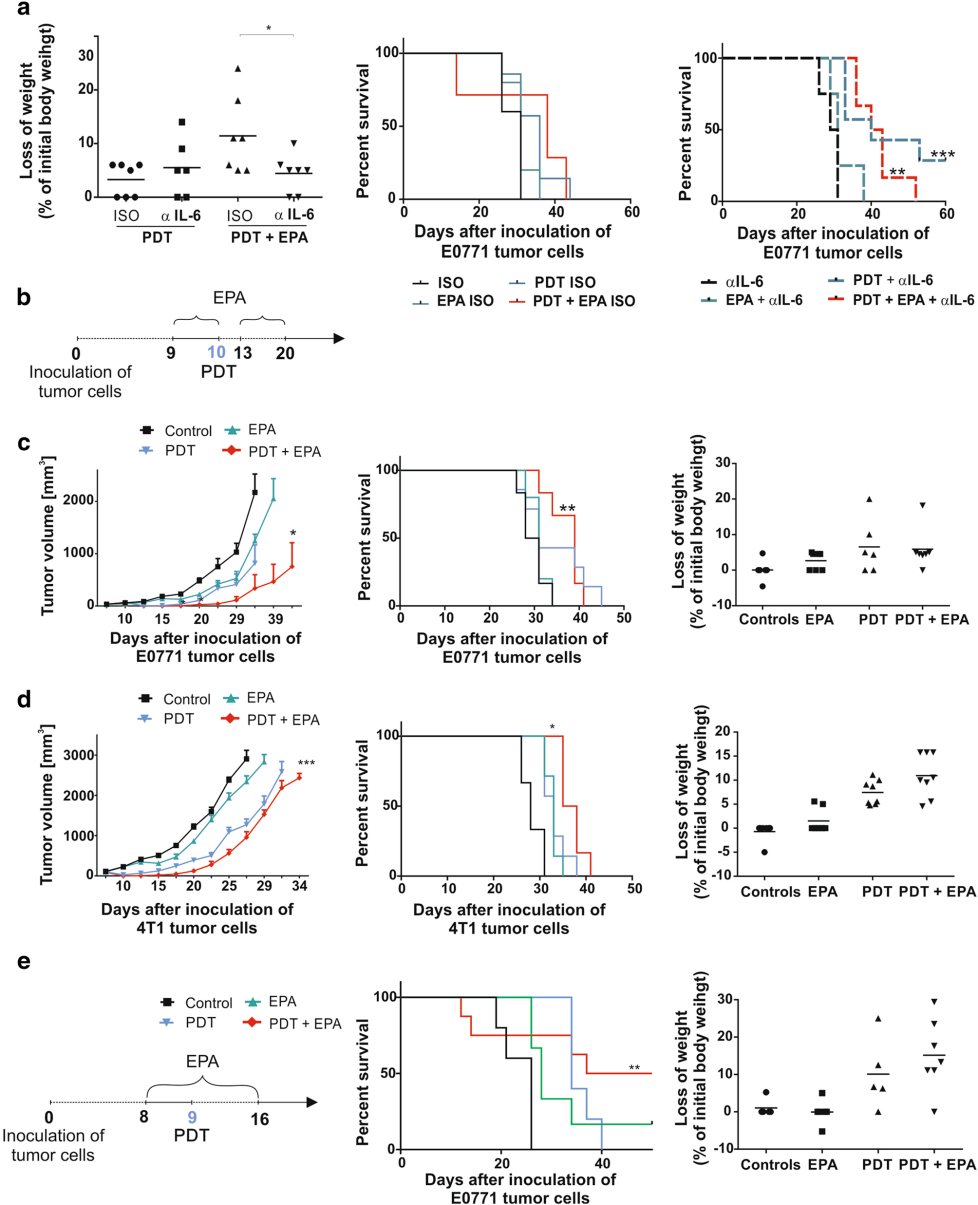
Effect of the PDT, EPA and anti-IL-6 antibodies combination treatment. **a** Experiment was carried out as shown in Fig. 2b. The anti-IL-6 or appropriate isotype control antibodies were administered i.p. at a dose of 100 µg per mouse, every second day, in 6 doses, starting from 1 day before PDT. Loss of weight (left) presented as percent of initial weight. Data present individual values with the means (bars), *n* = 6–7; *P < 0.05. The graphs represent Kaplan–Meier plots of the survival of mice from all experimental groups with isotope control (middle) or anti-IL-6 (right) antibodies, *n* = 6–7; *P < 0.05. **b** Detailed new experimental scheme. **c**, **d** Left panel shows mean tumor volumes ± SEM, middle panel shows corresponding Kaplan–Meier survival plots and right panel presents loss of weight of mice-bearing E0771(C) and 4T1(D) tumors in all experimental groups, *n* = 6–8; *P < 0.05; ***P < 0.005. **e** Left panel shows detailed experimental scheme of intratumoral EPA administration, middle panel shows Kaplan–Meier survival plots and right panel presents loss of weight of mice-bearing E0771 tumors, *n* = 5–7; **P < 0.05

IL-6 neutralization was shown to ameliorate systemic inflammatory adverse effects of various immunotherapies [24]. We observed that IL-6 neutralization with anti-IL-6 antibodies protected mice from bodyweight reduction and diminished the lethal effects of the combined PDT + EPA treatment (Fig. 4a, left). Although IL-6 neutralization significantly prolonged survival of mice treated with PDT, the antitumor efficacy of combined PDT + EPA treatment was abolished and comparable to the effect obtained in PDT-only group (Fig. 4a, middle and right).

Additionally, to avoid excessive toxicity we modified the treatment protocol with EPA in combination with PDT. To this end, EPA was administered in two rounds: on days 9 and 10, i.e., before PDT and after 3 days of rest; when the acute PDT-induced inflammation becomes attenuated, it was continued on days 13–20 (Fig. 4b). In this therapeutic scheme, EPA significantly potentiated PDT-induced tumor growth retardation in E0771 tumor model and prolonged mice survival (Fig. 4c). Antitumor efficacy of this combination treatment was similar in 4T1 tumor model, where PDT + EPA or PDT + EPA-analogue also significantly inhibited tumor progression and prolonged survival (Fig. 4d and Supplementary Fig. 2e and 2f). Finally, EPA was also administrated intratumorally. This approach revealed that although EPA significantly prolonged the survival of PDT-treated mice, it also caused toxic reactions and reduced mice weight, but only when combined with PDT (Fig. 4e).

## Discussion

PDT can be an effective and minimally invasive strategy to treat different types of superficial early-stage tumors without radiation and large incisions. In advanced metastatic tumors, it is rather a palliative treatment that can be used supplementary to surgery or optionally as an organ-sparing treatment [25]. PDT leads to tumor cell death accompanied by extensive oxidative stress and induction of local inflammation [26]. Both preclinical and clinical studies demonstrated that PDT through the induction of innate immune response is capable of activating adaptive immune response against tumors [27, 28]. This feature makes PDT increasingly more attractive treatment option, which can be potentially used in combination with cancer immunotherapies. However, in experimental tumor models, the complete antitumor responses to PDT are limited to some particular conditions when the development of immune response is facilitated by the use of highly immunogenic, carcinogen-induced tumors or tumors that are derived from genetically modified cells that express strong tumor-associated antigens [27–29]. Hence, it is widely discussed that PDT, apart from robust inflammation, induces certain compensatory mechanisms that limit the development of tumor-specific adaptive immunity [12, 30]. Not many studies have focused on immune evasion-associated events during or after PDT. For example, PDT was shown to induce expansion of Treg and increased secretion of IL-10 and TGF-β [11].

Here, we identify IDO as an immunoregulatory enzyme induced by PDT within tumors as well as in TDLNs. The obtained results suggest that the major source of IDO in the tumor microenvironment are granulocytic CD11b^+^Ly6G^+^ myeloid cells that strongly infiltrate the tumor after treatment and reveal the highest expression levels of this enzyme. Moreover, monocytic CD11b^+^Ly6C^+^ myeloid cells, although less abundant after PDT, upregulate IDO and might also play an important role in immunoregulation. The conclusion can be inferred from ex vivo studies, showing that CD11b^+^ cells isolated from PDT-treated tumors suppress proliferation of T cells more effectively as compared with CD11b^+^ cells isolated from control tumors. Although the Ly6G^+^ myeloid cells revealed the immunosuppressive potential, they were also shown to play a crucial role in the induction of long-term antitumor immune response after PDT [31]. Therefore, further mechanistic studies should be introduced to reveal the role of neutrophils subpopulations in PDT-mediated inflammation.

Increase in IDO activity after the PDT treatment is accompanied by the rise in the percentage of Treg in TDLN. It was previously shown that PDT increases the number of Treg in mice and the removal of Treg is associated with improved antitumor efficacy of PDT [11]. Here we show that IDO inhibition with EPA brings back the number of Treg to control values indicating potential involvement of this enzyme in PDT-induced Treg expansion. Kynurenic acid, a direct product of tryptophan degradation catalyzed by IDO, was shown to induce Treg expansion, by activating aryl hydrocarbon receptors [32].

Considering a number of immunoregulatory mechanisms associated with IDO activity, the selective inhibitors of this enzyme have been developed and progressed to clinical trials. IDO is an important immunoregulatory enzyme that evolved to control exuberant immune response mitigating tissue damage and immunopathology.

We observed exaggerated toxicity of orally applied EPA that evolved directly after PDT. Moreover, injection of EPA intratumorally prolonged the survival of PDT-treated mice what is in line with observation done by Lu et al. [33]. Importantly, in our experimental settings the local administration of EPA did not protect completely from toxic effects. Acute inflammation was associated with IL-6 release and massive infiltration of granulocytic myeloid cells (CD11b^+^Ly6G^+^) to the tumor bed as well as to the TDLNs. The toxic reaction was not reported in the studies where depletion of Treg was combined with PDT [11]. Nevertheless, increased concentrations of IL-6, as well as TNFα and IL-12, were previously described in response to simultaneous IDO inhibition and administration of apoptotic cells [34]. Inhibition of this enzyme resulted in the loss of self-tolerance to apoptotic cell-associated antigens and susceptibility to lupus-like autoimmunity. These data indicate that IDO plays an important role in the regulation of immune tolerance to antigens released from dying cells and suggest that PDT-induced tumor cells death may be one of the triggers leading to increased immunopathology.

IL-6 was previously shown to be induced by PDT in vitro and in vivo but also in cancer patients [31, 35]. The reports on its impact on the antitumor efficacy of PDT were discordant showing either negative [36], positive [37] or no effect [38]. Systemic inflammation can be ameliorated by administration of IL-6-neutralizing antibodies that are clinically validated in the management of immune-mediated adverse events, developing after cancer immunotherapies with checkpoint inhibitors or adoptive treatment with chimeric antigen receptor (CAR) T cells [39, 40]. Although IL-6 neutralization significantly potentiated antitumor efficacy of PDT, it eliminated the additional benefit of IDO inhibitor to the treatment. It is possible that IL-6 neutralization is associated with decreased IDO expression, as was shown in the previous study [41], that would tuck away the target for EPA making the treatment superfluous. Intriguingly, and in contrast to our observations, several recent studies have shown that combined blockade of IL-6 and PD-1/PD-L1 checkpoint molecules promotes tumor infiltration of IFN-γ-producing CD4^+^ T cells and exerts synergistic antitumor effects [42, 43].

Although IDO is an attractive target for cancer immunotherapies, its role in the regulation of inflammation remains not completely understood. Importantly, the promising results obtained with IDO inhibitors in mouse tumor models have not been successfully translated into the clinical trials. Current results are rather disappointing as IDO inhibition did not improve the immunotherapy of cancer. These failed clinical trials with IDO inhibitors underlined the complexity of tryptophan metabolism. The tryptophan can be degraded not only by IDO but also by tryptophan-2,3-dioxygenase or can be introduced into the serotonergic pathway, which products have also immunosuppressive properties [44]. On the other hand, in light of the success achieved by immunotherapies restoring the antitumor functions of T cells, studies on IDO inhibition provide a strong rationale for therapeutic targeting of this enzyme. Based on our results, we postulate to further elucidate the role of IDO in the systemic immune response to avoid unexpected acute reactions.

## Electronic supplementary material

Below is the link to the electronic supplementary material.

Supplementary material 1 (PDF 420 kb)

## Abbreviations

Arg1: Arginase 1
EPA: Epacadostat
iNOS: Inducible nitric oxide synthases
PDT: Photodynamic therapy
ROS: Reactive oxygen species
