# Supplementary material for: Inhibition of IDO leads to IL-6-dependent systemic inflammation in mice when combined with photodynamic therapy
Source: Cancer Immunol Immunother. 2020 Feb 28;69(6):1101–12. doi: 10.1007/s00262-020-02528-5 (PMC7230067; doi:10.1007/s00262-020-02528-5)
Supplement: Supplementary file 1 — Supplementary material 1 (PDF 420 kb) [file 262_2020_2528_MOESM1_ESM.pdf]

Supplementary materials

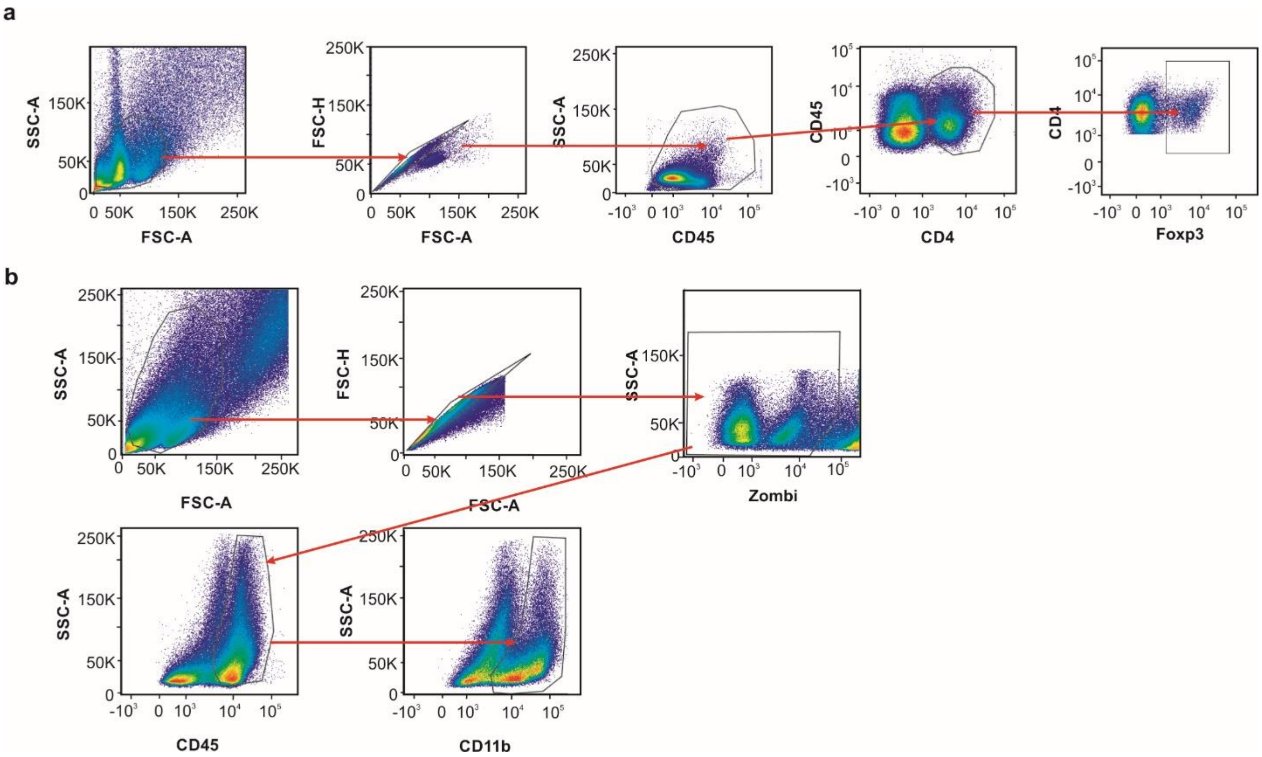

Supplementary Figure 1. a. Gating strategy for Treg cells (CD45$^+$CD4$^+$Foxp3). **b**. Gating strategy for tumor associated immunological CD11b$^+$ cells.

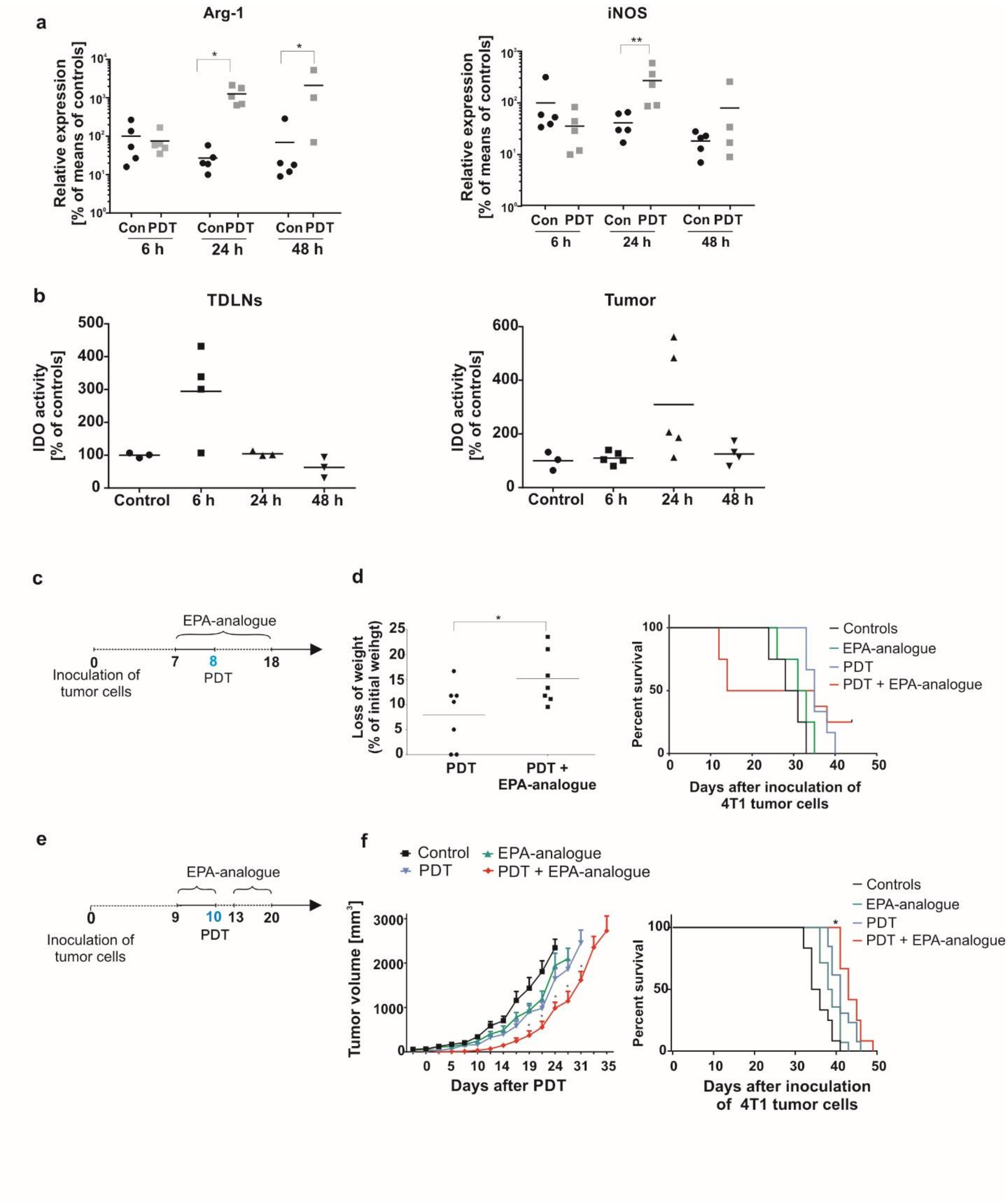

Supplementary Figure 2. a. Expression of Arg-1 and iNOS in E0071 tumors**.** Expression was measured in tumors 6, 24 and 48 h post PDT. mRNA levels were determined using real-time PCR. Data presents % of means of controls in experimental groups, n = 3-6, *P < 0.05. **P < 0.01 **b.** Activity of IDO enzyme evaluated in TDLNs (left) and 4T1 tumors (right) 6, 24 and 48 h post

PDT. Data presents % of controls in experimental groups, n = 3-5. **c**, **e.** Detailed experimental scheme. **d.** Left panel refers to weight loss of tumor bearing mice and right panel presents corresponding Kaplan–Meyer survival plots in all experimental groups, n = 6-8, *P < 0.05. **f**. Left panel shows mean tumour volumes ±SEM. Graphs present mean values from two independent repeats. Right panel presents corresponding Kaplan–Meyer survival plots in all experimental groups, n = 12-14, *P < 0.05
